# Dynamic Evolution and Spatial Convergence of the Virtual Cultivated Land Flow Intensity in China

**DOI:** 10.3390/ijerph18137164

**Published:** 2021-07-04

**Authors:** Kunpeng Wang, Wenjun Wu, Awais Jabbar, Zinabu Wolde, Minghao Ou

**Affiliations:** 1 College of Land Management, Nanjing Agricultural University, Nanjing 210095, China; 2018209019@njau.edu.cn (K.W.); 2019209014@njau.edu.cn (W.W.); Awaisjabbar37@gmail.com (A.J.); sos.zine04@gmail.com (Z.W.); 2 Center of Urban-Rural Joint Development and Land Management Innovation, Nanjing 210095, China

**Keywords:** virtual cultivated land, flow intensity, ecological compensation, convergence, dynamic evolution

## Abstract

Exploring the flow intensity of virtual cultivated land is the key to improving the ecological compensation and food security policy. This study aimed to analyze the dynamic evolution, spatial convergence, and its affecting factors of the virtual cultivated land flow intensity. The spatial convergence model was used in this study. The results showed that, during 2000–2018, the growth rate of the surplus state of virtual cultivated land at the national level is less than that of the deficit state of virtual cultivated land in China. Moreover, the number of deficit provinces of virtual cultivated land flow intensity is increasing. The absolute β-convergence characteristics of the virtual cultivated land flow intensity are significant at the national, northeast, central, and western regions. Additionally, the conditional β-convergence exists at the national and four regional levels. Meanwhile, cultivated land resource endowment, population scale, regional economic development level, and agricultural mechanization level play an important role in the convergence process of inter-regional virtual cultivated land flow intensity. However, the influence degree of different control variables on different regional virtual cultivated land flow intensity is not consistent. Therefore, policymakers should pay attention to cultivated land resources’ spatial transfer mechanism when making regional cultivated land ecological compensation policies to coordinate the interesting relationship between the deficit area and surplus area of virtual cultivated land. Therefore, it is necessary to take the virtual cultivated land flow intensity as the reference index and use the combination of market guidance and government control to stimulus the stakeholders to protect cultivated land by taking different measures.

## 1. Introduction

Cultivated land resources are an essential material basis for human survival and necessary strategic resource for social and economic growth, which has been observed worldwide [1,2]. The reason is that cultivated land has basic food production function [3] and has ecological functions such as regulating climate, maintaining soil and water, maintaining biodiversity, and so on [4,5,6]. It means that cultivated land can produce economic value [5] and produce ecological value [7]. Even the cultivated land’s environmental value accounts for a large proportion of cultivated land’s total value [8]. However, due to the environmental importance of cultivated land belonging to public goods [8], which has externality characteristics [9], the value cannot be traded in the market in the form of physical objects [10], so the cultivated land’s ecological value has been ignored by people [11]. What is worse, in recent years, with the rapid development of industrialization and agriculturalization in developing countries, cultivated land has been used irrationally, and polluted and damaged seriously, which has caused a lot of cultivated land ecological problems [5]. To protect the cultivated land ecological value [12] and realize the sustainable development of cultivated land resource [13], many countries are implementing the ecological compensation policy for cultivated land [14]. Since 2008, the Chinese government has selected Chengdu City in Sichuan Province as the experimental area of cultivated land ecological compensation mechanism, which is a substantial exploration of cultivated land ecological compensation policy.

The accounting of the cultivated land environmental value is the key to implementing the ecological compensation mechanism of cultivated land [15,16]. At present, the calculation methods of cultivated land ecological value mainly include the Contingent valuation method [17], Ecosystem service value method [18], Cost-benefit method [19], etc. Although these methods all consider the ecological value of cultivated land, the disadvantage of these calculation methods is that cultivated land value is too high to effectively guide the cultivated land ecological compensation [20,21]. Another one of the main problems is that the cultivated land ecological compensation policy is difficult to be fully implemented in the experimental area. Thus, although scholars have been trying to explore the calculation method of cultivated land ecological value [22,23], there are still some shortcomings in solving this problem [24,25].

The virtual cultivated land becomes a new perspective to calculate the cultivated land value [26,27]. The virtual cultivated land is put forward based on the concept of the virtual earth [28]. The connotation of virtual cultivated land is that cultivated land resources are “hidden” in a specific product or service [29], making the cultivated land resources move in the spatial area. Most of the studies on virtual cultivated land mainly focus on the impact of virtual cultivated land on the import and export trade of agricultural products [30,31], and quantitative measurement of virtual cultivated land [32], even the impact on the economy [33]. Virtual cultivated land strategy breaks through the space limitation of cultivated land resource flow and promotes the rational allocation of cultivated land resources in the country or region [34]. These studies have contributed significantly to assess the cultivated land ecological value. China’s land area is relatively large. The characteristics of cultivated land resources, the level of agricultural mechanization, land use management policies and so on in different regions show obvious differences, which leads to the differences in the spatial flow of virtual cultivated land in the different areas, and finally, in the form of the virtual cultivated land flow intensity. However, there is still a lack of research on the spatial difference and dynamic evolution of virtual cultivated land flow intensity in China.

What are the differences in the flow intensity of virtual cultivated land among different regions? Will the difference change over time? What are the main influencing factors? With consideration of the above problems, the data of 31 provinces in mainland China during the period 2000–2018 are utilized to investigate the dynamic evolution law and spatial differentiation characteristics of virtual cultivated land flow intensity. Furthermore, the convergence models are used to analyze the existence of convergence. This study is intended to provide a theoretical basis for the theoretical application of virtual cultivated land in cultivated land ecological compensation. At the same time, it can provide theoretical and practical reference for the government to solve the problem of unfair distribution of agricultural resources in different regions. According to the purpose of this study, the paper is organized as follows. The first section presents the brief introduction. Sequentially, Section 2 presents the virtual cultivated land flow intensity accounting framework and spatial convergence model. The data used in the analysis and the flow intensity of virtual cultivated land and convergence results are discussed in Section 3. The final Section 4 and Section 5 conclude the discussion and the main conclusions from this work and raise the research’s relevant political recommendations.

## 2. Materials and Methods

### 2.1. Virtual Cultivated Land Flow Intensity Accounting Framework

The concept of virtual cultivated land flow intensity refers to the ability of net outflow and net input of virtual cultivated land in different regions [29]. In fact, the flow of grain between provinces also drives the flow of virtual cultivated land [32]. Therefore, this study analyzes the flow of virtual cultivated land, and determines the corresponding surplus and deficit areas of virtual cultivated land. Specifically, if the total grain output of the province is greater than its own consumption, it indicates that there is a virtual cultivated land surplus in the province [35]. If the total grain output of the province is less than its own consumption, there is a virtual cultivated land deficit in the province. The virtual cultivated land flow intensity is measured by net surplus or deficit of regional virtual cultivated land to sown area [32]. Starting from the state of facts above, the research addressed, in detail, the issue of virtual cultivated land in regional pattern, based on several indicators viewed as significant for the assessment of flow intensity in virtual cultivated land. This study first analyzed the content of virtual cultivated land in grain products per unit mass. The virtual cultivated land area of grain products per unit mass can be defined as:

(1) $$CVLC_{i}=SFC_{i}/TFC_{i}$$

Equation (1) is based on prior literature [29,32,36]. *CVLC_i_* is the virtual cultivate land content of grain products per unit mass (unit: m^2^/kg); *SFC_i_* is the sown area of food crops in region *i* (unit: m^2^). In the *National Bureau of Statistics*, grain crops include cereals (rice, millet and corn), beans and potatoes, because these crops are the staple food of the Chinese people. Therefore, the data analysis is representative. Moreover, the grain crop multiple cropping index has been considered in the grain crop sown area published in the *Statistical Yearbook*; *TFC_i_* represents the total output of food crops in region *i* (unit: kg), which is the sum of cereals (rice, millet and corn), beans and potatoes of each province.

The imbalance of grain output and consumption among regions is the direct driving force of grain flow [29,32]. It shows that the virtual cultivated land will move with the grain circulation, and the flow of virtual cultivated land generally flows from surplus regions to deficit regions. Based on the virtual cultivated land content of grain products per unit mass, the number of regional virtual cultivated land surplus or deficit is the output of food crops in region i minus its consumption [37]. The Equation can be showed as:

(2) $$NF_{i}=TFC_{i}-CFC_{i}$$

In Equation (2) [35,36], *NF_i_* is the number of regional virtual cultivate land surplus or deficit (unit: kg). According to this index, the surplus or deficit of virtual cultivated land in different regions can be determined. Moreover, the surplus regions of virtual cultivated land are the compensated regions of ecological compensation of cultivated land. The deficit regions of virtual cultivated land are the payment regions of cultivated land ecological compensation. *CFC_i_* is the consumption of food crops in the region (unit: kg). *CFC_i_* is calculated by multiplying the national per capita consumption of food crops and the regional population. As there is a large number of flowing population in China, the population in this study refers to the permanent population in each province.

Based on the provincial data, the ratio of the surplus or deficit content of virtual cultivated land to the content of virtual cultivated land per unit grain quality is used to measure the net surplus or net deficit product of virtual cultivated land in each province. Thus, the Equation can be showed as:

(3) $$NF_{0}=NF_{i}/CVLC_{i}$$

In Equation (3), this equation is based on five authors own illustration drawn from the studied empirical and theoretical literature. *NF_0_* is the net surplus or deficit of the virtual cultivated land in the region (unit: kg). The indicator describes the area of net surplus or net deficit of virtual cultivated land contained in different regions. According to this index, the net outflow and net input of virtual cultivated land in different regions can be determined.

Based on the net surplus or deficit of the virtual cultivated land in the region, the ratio of net surplus or net deficit of virtual cultivated land to sown area of grain crops in different provinces is used to measure the flow intensity of virtual cultivated land. Thus, the equation can be showed as:

(4) $$K=NF_{0}/SFC_{i}$$

In Equation (4), this equation is based on five authors own illustration drawn from the studied empirical and theoretical literature. *K* represents the virtual cultivated land capacity of net outflow and net input of virtual cultivated land in different regions. According to the index *K*, the virtual cultivated land capacity of net outflow and net input in different regions can be determined.

### 2.2. The Spatial Convergence Model

In general, it is found that σ-convergence, absolute β-convergence and conditional β-convergence are the most representative methods for convergence analysis [36]. Spatial convergence analysis can explore the “convergence” or “divergence” of the virtual cultivated land flow intensity within a region. Suppose there is a convergence characteristic in the flow intensity of virtual cultivated land in mainland China. In that case, it indicates that the gap is narrowing in the flow intensity of virtual cultivated land in each region [38], reflecting the increase in food production capacity in the area as a whole [39]. Conversely, it shows that the gap is expanding in the flow intensity of virtual cultivated land in each region, which means a decline in regional food production capacity.

#### 2.2.1. σ-Convergence

σ-convergence is mainly used to describe the stock level of virtual cultivated land in each region [40]. When there are objective differences in natural resource conditions in the different areas, σ-convergence can test the temporal changes of the flow intensity of virtual cultivated land across the country and various provinces. This study used the standard deviation analysis method to test σ-convergence [41]. Standard deviation is an index to measure the degree of variation of a variable [42]. The greater the value of the standard deviation, the greater the variable’s degree of variation, and the lower the representativeness of the average [43]. This method’s advantage is that it can directly reflect the deviation of the sample gap from the mean. The calculation formula of the σ-convergence model is as follows:

(5) $$\sigma_{t}=\sqrt{\left[\sum_{i=1}^{n}{\left({\mathrm{lnG}}_{it}-\bar{{\mathrm{lnG}}_{t}}\right)}^{2}\right]/N}$$

In Equation (5) [44], lnG*_it_* is the logarithm of the virtual cultivated land flow intensity of province *i* in *t* year. $\bar{\ln G_{t}}$ is the average logarithm of the virtual cultivated land flow intensity in each region in year *t*; *N* is the number of provinces.

#### 2.2.2. Absolute β-Convergence

The absolute β-convergence is mainly used to analyze whether the growth rate of virtual cultivated land flow intensity in each province converges [36,38]. However, absolute β-convergence is based on the assumption that there is no difference in social, economic and resource conditions of regions [45,46]. To analyze whether there is a catch-up effect in the flow intensity of virtual cultivated land in each province, whether the gap in the flow intensity of virtual cultivated land in each province is narrowed, and finally tending to the same stable state [47], the calculation formula of absolute β-convergence is as follows:

(6) $$\frac{1}{T}\ln\left(\frac{P_{i,t+T}}{P_{i,t}}\right)=\alpha +\beta_{i}\ln(P_{i,t})+\lambda_{i,t}$$

In Equation (6) [36,37,38,39,47], *i* represents each province or region; *P_i,t+T_* represents the surplus or deficit of virtual cultivated land in province *i* in the period (*t* + *T*); *P_i,t_* represents the surplus or deficit of virtual cultivated land in province i in the period t; α is the constant intercept terms; *β* is the regression coefficient. *λ_i,t_* is the random error terms. If *β* value is significant and *β* < 0, it means that there is absolute β-convergence, and the growth rate of the flow intensity of virtual cultivated land is inversely proportional to the initial level, indicating that provinces with relatively lagging flow intensity tend to catch up with regions with relatively high flow intensity.

#### 2.2.3. Conditional β-Convergence

The conditional β-convergence eliminates the assumption that there is no difference in social, economic and resource conditions [48]. The condition β-convergence adds some control variables [49], such as regional resources, social environment, economic development level, etc. The model is mainly used to test whether the flow of virtual cultivated land in domestic and regional areas will converge to different stable state according to its own convergence path. Generally speaking, the common method to verify conditional convergence is the conditional β-convergence method, which is an important supplement to the absolute β-convergence [45]. The mathematical expression of conditional β-convergence is as follows:

(7) $$\frac{1}{T}\ln\left(\frac{P_{i,t+T}}{P_{i,t}}\right)=\alpha +\beta_{i}\ln(P_{i,t})+\mu X_{i,t}+\lambda_{i,t}$$

In Equation (7) [49], *X_i,t_* represents the control variables; *μ* is the estimated coefficient of the control variable *X*, and the meaning of other variables is the same as Equation (6). If *β* value is significant and *β* < 0, it indicates a condition of *β* convergence, that is, convergence to its stable level. In addition, conditional β-convergence is compared with conditional β-convergence [45,48,49]. The only difference between conditional β-convergence is the addition of control variables, enhancing the ability to explain regional differences in specific geographic phenomena [49].

### 2.3. Data

#### 2.3.1. Study Area

**Figure 1:**
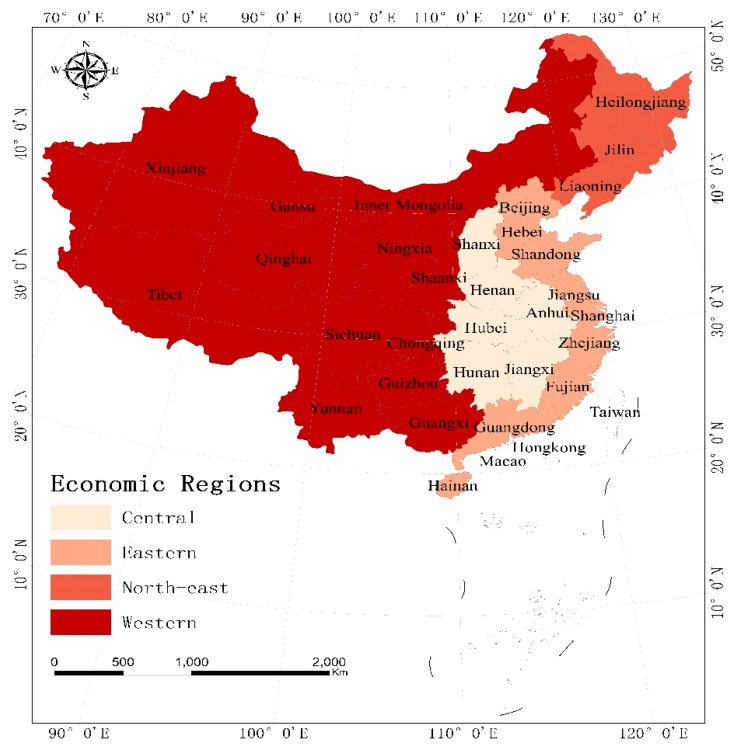
Map of the research area.

In this study, the virtual cultivated land flow intensity assessment is conducted on only 31 of the 34 province regions of China (Self-administered districts, Municipalities) (Figure 1) due to the lack of data about Hong Kong, Macau and Taiwan. Thus, this study does not include Hong Kong, Macau and Taiwan regions in China. According to the social and economic development of different areas of China, from the perspective of policy, the Chinese government divides the country into four economic regions: the eastern region, the northeast region, the central region, and the western region. From 2000 to 2018, the total sown area of grain increased from 10,846.3 × 10^4^ ha to 11,703.8 × 10^4^ ha [50], and the total yield of grain crops increased from 46,217.5 × 10^4^ t to 65,789.2 tons × 10^4^ t. However, there are significant differences in China’s cultivated land resources in the spatial area [51].

The cultivated land resource in the eastern region is 2629.7 million hectares, accounting for 19.4% of the country’s cultivated land resources [50]. The eastern region is endowed with a suitable natural environment suitable for intensive crop cultivation [51]. The area also had a high population density among other regions. The population of the eastern region accounts for 41.2% of the country’s population [50]. The cultivated land resource in the central region is 3071.5 million hectares, accounting for 22.7% of the country’s cultivated land resources, while the population of the central region accounts for 26.5% of the country’s population.

Furthermore, the cultivated land resources in the western region and northeast region are 5043.5 million hectares and 2793.8 million hectares, accounting for 37.3% and 20.6% of the country’s cultivated land resources. Besides, the cultivated land resources in the central and western regions are mainly dryland. The western region had a low population density among other regions.

#### 2.3.2. Data Sources

The data is based on the provincial area. This research mainly considers grain types such as grains, potatoes, and beans to calculate the inter-provincial virtual cultivated land flow intensity, and finally, calculate the cultivated land flow intensity at the regional level. This study’s economic and social data mainly come from the *China Statistical Yearbook*, *China Demographic Yearbook*, and the national economic and social development bulletins of various provinces. Besides, to eliminate the impact of price changes and uniformly calculate the economic data, the standard is adopted to convert the corresponding price index into a constant price in 2000. The purpose is to help compare the dynamic evolution law of the virtual cultivated land flow intensity between regions. It can better compare the spatial convergence characteristics of the virtual cultivated land flow intensity.

### 2.4. Statistical Analysis

In this study, the data analysis is conducted in three parts: (1) Calculating the surplus or deficit of virtual cultivated land of study area during the period 2000–2018; (2) Calculating the virtual cultivated land flow intensity in inter-provincial and mainland China. Then, to analyze the dynamic evolution law during the period 2000–2018. Besides, the spatial processing ability of GIS is used to express the data and highlight the regional differentiation law of virtual cultivated land flow intensity; (3) Calculate and compare the σ-convergence, absolute β-convergence, and conditional β-convergence using Stata 14.0 software.

## 3. Results

### 3.1. Dynamic Evolution of Virtual Cultivated Land Flow Intensity

**Figure 2:**
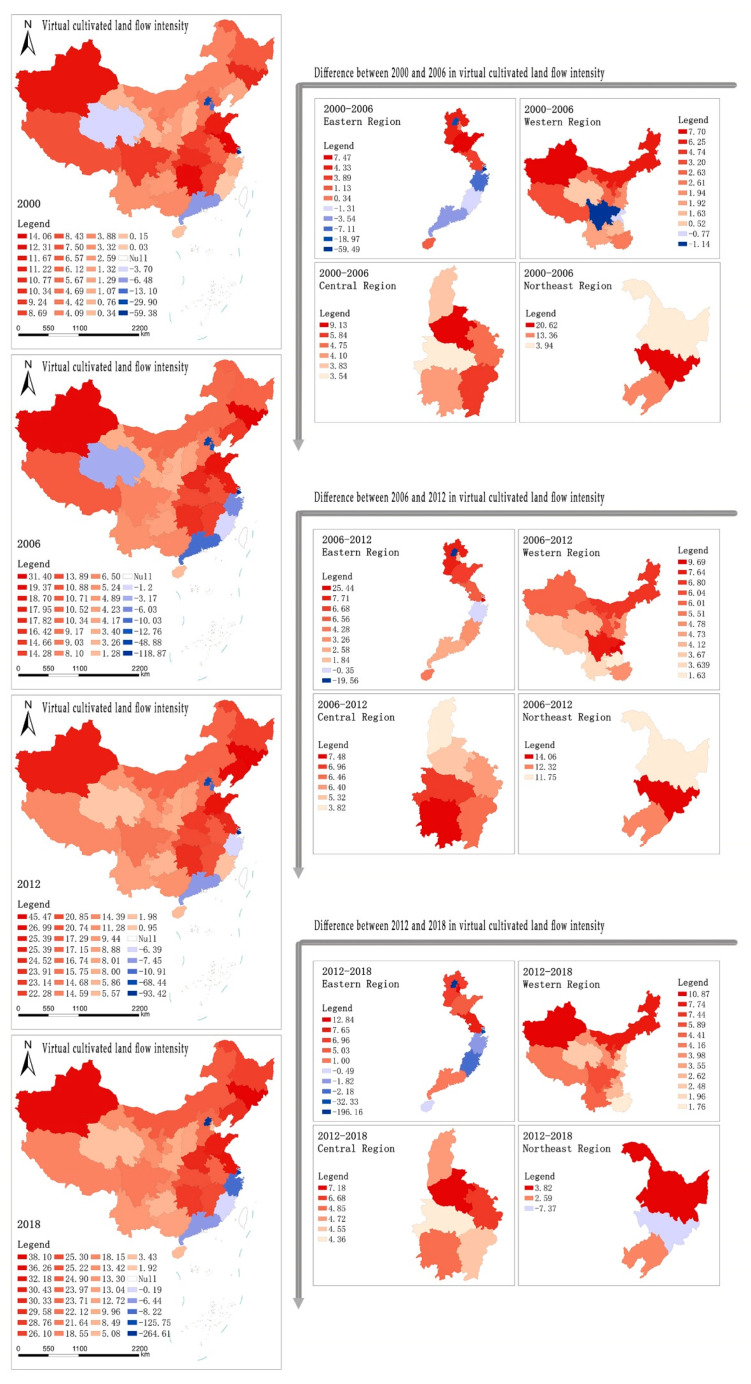
The distribution pattern of virtual cultivated land flow intensity in mainland China (Notes: The figure on the left shows the virtual cultivated land flow intensity in 31 provinces of China from 2000 to 2018, with red representing the surplus area and blue representing deficit area, and the intensity of surplus/deficit expressed by color concentration, that is, the darker the color, the greater the intensity; the right figure is a regional diagram of the difference of virtual cultivated land flow intensity in different periods, and the red and blue are used to distinguish increase and decrease. Similarly, the degree of increase/decrease is expressed by color depth.).

There are surplus status and deficit status in the virtual cultivated land flow intensity in this study. Figure 2 shows the changes in the virtual cultivated land flow intensity during the period 2000–2018 in this study area. Some results are shown: (1) At the national level, the number of deficit provinces of virtual cultivated land flow intensity is increasing during the period 2000–2018, and these provinces are mainly distributed in the eastern coastal areas of China; (2) To the deficit status of virtual cultivated land flow intensity, the maximum value of the virtual cultivated land flow intensity is 59.38 in 2000, and its maximum value is 264.61 in 2018. To the surplus status of virtual cultivated land flow intensity, the maximum value of the virtual cultivated land flow intensity is 14.06 in 2000, and its maximum value is 38.10 in 2018. It indicates that, during the period 2000–2018, the growth rate of the surplus state of virtual cultivated land at the national level is less than that of deficit state of virtual cultivated land.

From the perspective of regional division (Figure 2), from 2000 to 2018, the difference of virtual cultivated land flow intensity among the three provinces in the northeast region is gradually narrowing. However, in the study period, the difference of virtual cultivated land flow intensity in Jilin Province is the largest. The results show little difference in the virtual cultivated land flow intensity among the provinces in the central region. Among the provinces in the western region, the number of provinces with surplus status of virtual cultivated land flow intensity difference increases gradually. Still, the change of virtual cultivated land flow intensity difference is small.

### 3.2. Spatial Convergence of Virtual Cultivated Land Flow Intensity in China

#### 3.2.1. σ-Convergence Analysis

**Figure 3:**
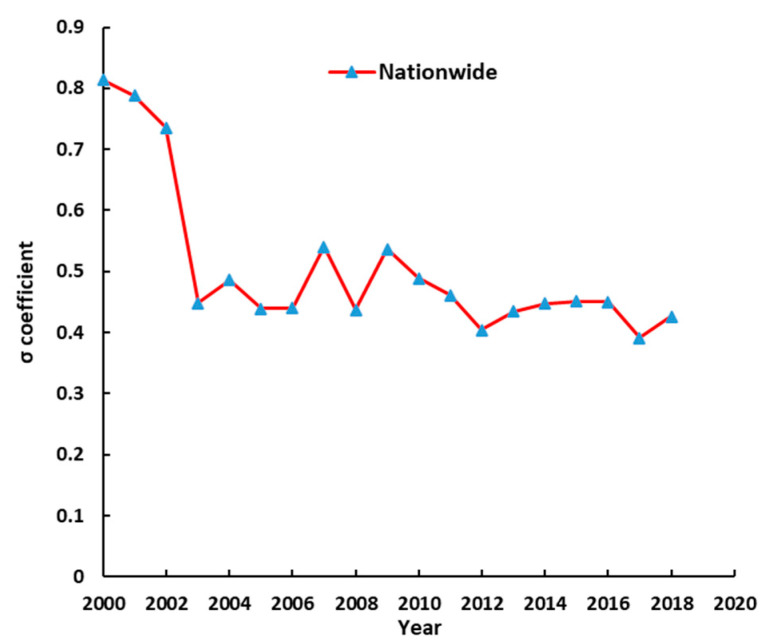
The σ coefficient changes and trends of the virtual cultivated land flow intensity nationwide.

To show the σ-convergence coefficient changes in virtual cultivated land flow intensity during 2000–2018 in the study area. Figure 3 displays the σ coefficient change trend of the virtual cultivated land flow intensity nationwide. According to the definition of σ-convergence model, if the time series curve of convergence coefficient shows a downward trend in a certain period, it indicates that there is convergence characteristic in that period. Conversely, it exists as the divergence characteristic.

In Figure 3, the σ coefficient exists as a downward trend during the period 2000–2018. It indicates that there is a significant convergence characteristic in the virtual cultivated land flow intensity nationwide. During 2000–2003, the σ coefficient has a significant downward trend. Additionally, the coefficient decreased from 0.8137 in 2000 to 0.4484 in 2003. The results indicate that the regional difference of virtual cultivated land flow intensity is gradually reduced during 2000–2003. Existing fluctuation period is of the σ coefficient during the period 2003–2012. However, the minimum σ coefficient during this period is lower than that during 2000–2003. Furthermore, the decline degree is smaller than that during 2000–2003. However, the σ coefficient displays a steady upward trend during the period 2012–2016. The coefficient increased from 0.4041 in 2012 to 0.4505 in 2016. However, the σ coefficient indicates V-shape in 2016–2018. Besides, the minimum σ coefficient of this study period is 0.3913 in 2017. Similarly, the σ coefficient displayed an upward trend in 2018; the result indicated that there exists an σ-divergence characteristic in the virtual cultivated land flow intensity in these regions in that period.

**Figure 4:**
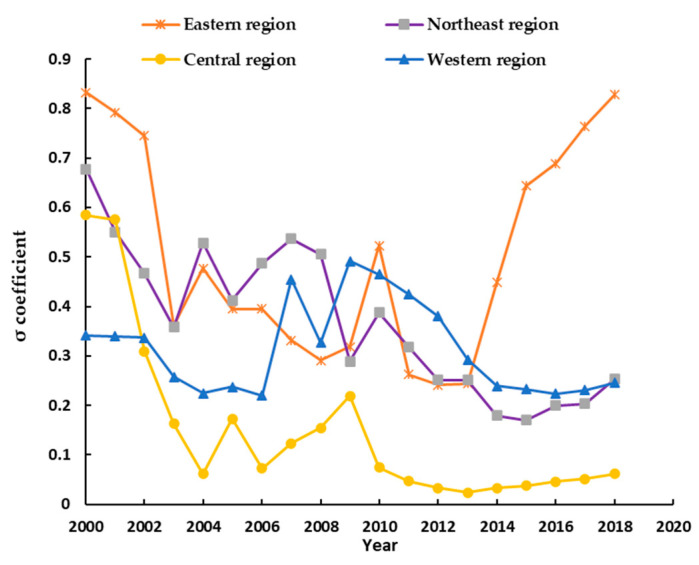
The σ-convergence change trend of the virtual cultivated land flow intensity in four major regions.

In Figure 4, from the σ coefficient of four regions, during 2000–2003, the σ coefficient exists a significant downward trend. It indicated a significant σ-convergence characteristic in the virtual cultivated land flow intensity in four regions [43]. It implied that the regional difference of the virtual cultivated land flow intensity is gradually reduced in four regions during the period 2000–2003.

Moreover, during the period 2000–2003, in the eastern region, central region, northeast region, and western region, the σ coefficient change degree is 0.4709, 0.4220, 0.3198 and 0.0838. Thus, the σ coefficient change degree order is eastern region > central region > northeast region > western region. The existing fluctuation period of σ coefficient is during the period 2003–2010. During the period 2010–2014, the σ coefficient displays the steady downward trend in the northeast region and western region. It indicated a significant σ-convergence characteristic in the virtual cultivated land flow intensity in four regions in that period [43]. However, after 2014, except the western region, the σ coefficient exists on an upward trend in other regions. Especially, the σ coefficient of the eastern region is on a significant upward trend. It implied that there is σ-divergence characteristic in the virtual cultivated land flow intensity in these regions in that period. However, after 2016, the σ coefficient of the eastern region is also on an upward trend.

#### 3.2.2. Absolute β-Convergence Analysis

In econometric analysis, to judge whether the panel data model is designed as fixed effect model or random effect model, the Hausman test is needed [30]. In theory, because the data in this paper are from 31 provinces, it cannot be regarded as random samples. In the empirical test, data were processed using Eviews 10.0 software by IHS Global Ins. of USA. Table 1 shows the results of Hausman Test, and the test results of the national and four regional models for absolute and conditional β-convergence analysis are included. According to Table 1, the model rejects the original hypothesis that individual influence is not related to the explanatory variable at the level of 1% significance, that is, the fixed effect model should be selected for both the cross-section model and the time model.

According to the results of the Hausman Test, this study uses a fixed-effect model to perform absolute β-convergence analysis for the nationwide and four major regions. Table 2 represents the results of the absolute β-convergence estimation model. From the results, it specifically illustrates significant positive linkages as follows:

Firstly, the β-convergence coefficient of virtual cultivated land flow intensity is both significant and negative in nationwide, as are the northeast, central region and western region. Moreover, their coefficients are all significant at the 1% confidence level. The results showed that there are absolute β-convergence characteristics in these regions. As time goes by, the spatial difference in the virtual cultivated land flow intensity shows a gradual shrinking trend [49]. It implied that there is the catch-up effect in backward areas. That is to say, the grain production capacity of regions with low flow intensity of virtual cultivated land has increased faster, and eventually, it will be consistent with regions with high flow intensity of virtual cultivated land. Secondly, from each region’s results, one of the most significant convergence tend in the northeast region, followed by the western region and central region. However, although the absolute β-convergence coefficient of virtual cultivated land flow intensity is negative in the eastern region, it is not significant. It means that there is no significant absolute β-convergence characteristic of the flow intensity of virtual cultivated land in the eastern region during the study period.

The absolute β-convergence model of the virtual cultivated land flow intensity of nationwide and four regions is passed by the significance testing. However, from the goodness of fit value of the model, the goodness of fit is between 0.002–0.246, indicating that the goodness of fit is low. Maybe the reason is, in fact, the virtual cultivated land flow intensity is greatly affected by regional economic, social and resource conditions [51]. However, absolute β-convergence is based on the assumption that there are no differences in social, economic, and resource conditions [47]. Therefore, when analyzing the convergence of the flow intensity of virtual cultivated land, the influence of control variables must be considered.

#### 3.2.3. Conditional β-Convergence Analysis

The temporal changes and spatial differences of virtual cultivated land flow intensity are closely related to regional natural resources, economic, social and policy factors. Based on absolute β-convergence analysis, some control variables need to be added when performing convergence analysis in this study. There are five control variables in the conditional β-convergence.

(1) Effective irrigation area (EIA) is one of the representative factors of land resource endowment. EIA is the sum of the area of paddy field and irrigated land which have been equipped with irrigation engineering or equipment and can be irrigated normally [52]. EIA is important as an index to measure the stability of agricultural production. The increase of EIA indicates that the land resource endowment is becoming better. If the spatial difference of land resource endowment is reduced, it indicates that the spatial difference of virtual cultivated land is reduced.

(2) Consumption of chemical fertilizers (CCF) is also selected as representative factors of land resource endowment. CCF has a positive effect on grain production [36], but the marginal effect of chemical fertilizer on grain production is decreasing [53].

(3) Regional population size (RPS) is selected as one of the control variables. People are not only producers of food, but also consumers of food. The larger the population scale is, the higher the grain consumption is [32,54], and the expansion of population scale will promote the increase of virtual cultivated land demand.

(4) Gross domestic product (GDP) represents the level of regional economic development. Agricultural output value is an important part of regional GDP [55]. The improvement of regional economic development level will increase the investment in agriculture, and the regional difference of agricultural output value will narrow, which indicates that the convergence trend of virtual cultivated land among regions will accelerate.

(5) Total power of agricultural machinery (RAM) reflects the level of agricultural modernization [56]. The higher the level of agricultural modernization, the greater the grain production capacity. The improvement of grain production capacity will promote the convergence trend of virtual cultivated land.

Table 3 displays the results of the conditional β-convergence estimation model. Firstly, on the whole, when some control variables are added in the model, the conditional β-convergence coefficients of the virtual cultivated land flow intensity are significant at national and four regional levels. Moreover, their coefficients are all significant. Furthermore, the model coefficients of all are negative at the national and four regional levels. The results showed that there is a significant characteristic of conditional β-convergence in these regions. Over time, the smaller spatial difference in the virtual cultivated land flow intensity in these regions exists. In addition, the convergence coefficient is −0.749 in the central region. It means that compared with the result of the nationwide, the convergence trend of the flow intensity of virtual cultivated land is the most obvious in the central region, followed by the northeast region and western region.

From the model results of various control (seen Table 3), this study found that the same factor has different effects on the flow intensity of virtual cultivated land among the different regions. This difference is mainly revealed in the direction and degree of influence.

From the result of the EIA, the variable coefficient is negative of the EIA in the western region and the variable coefficient is significant. Although the EIA coefficient is negative (−0.703), the relationship is not significant between the flow intensity of virtual cultivated land and the EIA. This means that there is a trend of conditional β-convergence in this region, but the result is not significant. From Table 2, the results of the central region in EIA coefficient are similar to those in the northeast region. However, conversely, in the eastern region, the EIA’s coefficient is positive, but it is not significant. On the one hand, from 2000 to 2018, the virtual cultivated land has been in deficit in the eastern region, and the deficit has been increasing year by year. On the other hand, in fact, there is too little cultivated land but a large population in the eastern region. The increase in EIA is much lower than the growth rate of regional population. Therefore, the eastern region is the highest level of economic development in China, and its regional characteristics are particularly crowded but cultivated land resources are particularly scarce. Moreover, this area is mainly based on the development of the tertiary industry, so EIA changes have little effect on the virtual cultivated land flow in this area.

From the CCF result, the variable coefficient is positive of the CCF nationwide and the other four regional levels. The results showed that CCF promotes the increase of the flow intensity of virtual cultivated land. Among them, in the central region and western region, the variable coefficients are significant. Moreover, the variable coefficients also are significant nationwide and in the eastern region. It indicates that CCF has a particularly significant effect on the promotion of the flow intensity of virtual cultivated land.

The coefficient of RPS is negative and significant in the eastern and western regions (seen Table 3); it indicates that RPS has an inhibitory effect on the convergence trend of the virtual cultivated land flow intensity in these two regions. The reason is that the urban population density and economic development level are relatively high in the eastern region, while the opposite is true in the western region. However, in the central region, the coefficient of RPS is 2.863; it implies that RPS has a significant role in promoting the flow intensity of regional virtual cultivated land.

In addition, it can be seen from the GDP results that the coefficients of GDP are positive and significant in the eastern, central and western regions, indicating that it has a significant role in promoting the flow intensity of regional virtual cultivated land in these regions. The PAM represents the level of agricultural mechanization. The coefficients of PAM are negative and significant in all regions. Table 3 shows that the level of agricultural mechanization has an inhibitory effect on the flow intensity of virtual cultivated land in various regions. Especially, in the northeast region, the results showed that the virtual cultivated land flow intensity decreased by 0.501% when PAM increases by 1%. Thus, the improvement of the level of agricultural mechanization may accelerate the convergence process among regions.

## 4. Discussion

Due to the research, the area is national level and provincial level, which means the study area is large-scale and the research period is long-scale; in the analysis of conditional β-convergence, the selected control variables have positive significance for the spatial evolution trend and convergence of large-scale cultivated land flow intensity. Due to the unpredictable abnormal climate, natural disasters and sudden public pandemic events (such as COVID-19), it may have an impact on the spatial distribution of small-scale virtual cultivated land [57]. Therefore, if the prediction of virtual cultivated land flow intensity is carried out at the small-scale level, more detailed influencing factors should be selected according to the natural, economic and social characteristics of the study area [58], and the threshold of virtual cultivated land flow intensity should be set [59].

This study shows that the influence degree of different control variables on different regional virtual cultivated land flow intensity is not consistent. Considering the evolution process will be affected by the regional nature resources, social economy and policy factors [51]; when the government adopts measures to stimulus the stakeholders of cultivated land protection for protecting cultivated land, it should pay attention to regional and spatial differences in cultivated land resource endowment, population scale, regional economic development level and agricultural mechanization level [60]. Such as, in the northeast, the level of agricultural mechanization has a significant inhibitory effect on the flow intensity of virtual cultivated land in various regions, which means that the improvement of the level of agricultural mechanization will accelerate the convergence process among regions.

In this study, from the perspective of virtual cultivated land, the surplus and deficit of virtual cultivated land in the national and provincial level was analyzed. In the process of implementing the policy of cultivated land ecological compensation in China, through the surplus and deficit of virtual cultivated land in space, we can identify the payer area and payee area of cultivated land ecological compensation [14], and then determine the compensation subject of cultivated land ecological compensation [34]. This study, which is based on the perspective of virtual cultivated land enriches the research perspective of ecological compensation of cultivated land, and is a better method to realize the internalization of externality of cultivated land ecological compensation value. However, at present, there is no perfect research method to internalize the external benefits of cultivated land ecological compensation [15], which is the fundamental reason for the low efficiency of cultivated land ecological compensation policy implementation [61]. Virtual cultivated land is a compelling exploration of cultivated land value identification, even if it is not perfect. In future research, based on the perspective of virtual cultivated land, the mechanism of cross-regional cultivated land ecological compensation, the standard of cultivated land ecological compensation, and the mechanism of cultivated land ecological compensation [62] are the further research direction in the future.

Cross-regional flow of virtual cultivated land will have different effects on the surplus area and deficit area [63]. Virtual cultivated land surplus area pays more attention to improving agricultural productivity [64], which will reduce the land supply of other industries, which is not conducive to the improvement of the comprehensive level of surplus area. For the virtual cultivated land deficit area, the inflow of virtual cultivated land provides the basic support for the deficit area’s economy [65]. However, excessive dependence on the inflow of virtual cultivated land will make the industrial structure of the virtual cultivated land deficit area unbalanced [63]. Therefore, to coordinate the interest relationship between the deficit area and surplus area of virtual cultivated land, the government should take the virtual cultivated land flow intensity as an essential factor in the establishment of the cultivated land ecological compensation mechanism.

## 5. Conclusions

In this study, the spatial convergence model analyzes the dynamic evolution and spatial convergence characteristics of the virtual cultivated land flow intensity in the national region and the four regions of eastern, central, western and northeast in China from 2000 to 2018. The main conclusions are as follows:

(1) At the national level, the number of deficit provinces of virtual cultivated land flow intensity increased during the period 2000–2018. These provinces are mainly distributed in the eastern coastal areas of China. Moreover, the growth rate of the surplus state of virtual cultivated land at the national level is less than that of the deficit state of virtual cultivated land.
(2) The σ coefficient showed a downward trend in 2000–2018, which shows that the spatial and regional differences of the virtual cultivated land flow intensity decrease with time.
(3) The absolute β-convergence characteristics of virtual cultivated land flow intensity are significant in the whole country, northeast, central and western regions. Conditional β-convergence exists at the national and four regional levels.
(4) Cultivated land resource endowment, population size, regional economic development level and agricultural mechanization level play an essential role in the convergence process of regional virtual cultivated land flow intensity. However, the impact of different control variables on the virtual cultivated land flow intensity in other regions is not consistent.

## Figures and Tables

**Table 1:** The results of the Hausman Test.

Items		Nationwide	Eastern Region	Northeast Region	Central Region	Western Region
Absolute β-convergence	Chi-Sq. Statistic	33.637418	13.854329	57.687461	69.854623	51.958637
	Prob.	0.0021	0.0000	0.0009	0.0015	0.0000
Conditional β-convergence	Chi-Sq. Statistic	46.754613	15.783945	49.517321	115.816894	53.842767
	Prob.	0.0000	0.0000	0.0017	0.0173	0.0000

**Table 2:** Absolute β-convergence results of the flow intensity of virtual cultivated land in China’s mainland area.

Variable	Nationwide	Eastern Region	Northeast Region	Central Region	Western Region
α	0.124 ***	0.093	0.386 ***	0.332 ***	0.276 ***
	(4.265)	(1.414)	(4.930)	(6.524)	(5.065)
β	−0.003 ***	−0.001	−0.116 ***	−0.015 ***	−0.018 ***
	(−2.964)	(−0.845)	(−4.244)	(−5.813)	(−5.890)
R^2^	0.015	0.005	0.246	0.231	0.096
Adjust R^2^	0.013	0.002	0.233	0.225	0.092
F-statistic	8.760	0.700	17.980	33.710	23.880
Samples	589	190	57	114	228

Notes: Significant at <1% (***); T-statistics in brackets.

**Table 3:** Conditional β-convergence results of the flow intensity of virtual cultivated land in study area.

Variable	Nationwide	Eastern Region	Northeast Region	Central Region	Western Region
α	−0.519 ***	18.172 **	−74.171	−34.353 ^***^	9.122
	(−14.860)	(1.714)	(−1.113)	(−2.921)	(1.327)
β	−0.183 ***	−0.472 ***	−0.682 ***	−0.749 ^***^	−0.598 ***
	(−5.801)	(−7.521)	(−7.560)	(−8.880)	(−10.091)
EIA	−0.171	0.209	−0.703	−0.567	−0.814 ***
	(−0.753)	(0.390)	(−1.010)	(−1.311)	(−2.051)
CCF	0.456 **	0.257 **	1.081	1.336 ***	0.458 ***
	(2.440)	(0.580)	(1.482)	(1.311)	(1.410)
RPS	−1.649	−2.401 **	9.142	2.863 ***	−0.901 ***
	(−3.193)	(−1.811)	(1.160)	(2.312)	(−0.930)
GDP	0.304	0.364 ***	−0.283	0.293 **	0.359 ***
	(4.380)	(1.850)	(−0.810)	(1.751)	(3.070)
PAM	−0.154 ***	−0.467 ***	−0.501 ***	−0.251 **	−0.018 ***
	(−0.970)	(−0.383)	(−0.860)	(−1.960)	(−0.061)
R^2^	0.113	0.120	0.544	0.310	0.161
Adjust R^2^	0.104	0.091	0.489	0.271	0.138
F-statistic	12.320	4.170	9.940	8.010	7.060
Samples	589	190	57	114	228

Notes: significant at <1% (***); 1–5% (**) levels of significance; T-statistics in brackets.

## Data Availability

Data available in a publicly accessible repository that does not issue DOIs Publicly available datasets were analyzed in this study. This data can be found here: http://www.stats.gov.cn/tjsj/ndsj/ (accessed on 2 July 2021).
